# Discussing prognosis and treatment goals with patients with advanced cancer: A qualitative analysis of oncologists’ language

**DOI:** 10.1111/hex.12549

**Published:** 2017-03-05

**Authors:** Wen‐ying Sylvia Chou, Lauren M. Hamel, Chan L. Thai, David Debono, Robert A. Chapman, Terrance L. Albrecht, Louis A. Penner, Susan Eggly

**Affiliations:** ^1^ National Cancer Institute Bethesda MD USA; ^2^ Wayne State University/Karmanos Cancer Institute Detroit MI USA; ^3^ Santa Clara University Santa Clara CA USA; ^4^ Josephine Ford Cancer Institute Detroit MI USA

**Keywords:** cancer, discourse analysis, patient‐provider communication, prognosis

## Abstract

**Background:**

The National Academy of Medicine recommends that cancer patients be knowledgeable of their prognosis to enable them to make informed treatment decisions, but research suggests few patients receive this information.

**Objective:**

This qualitative study describes oncologists’ language during discussions of prognosis and treatment goals in clinical interactions with African American patients diagnosed with cancer.

**Design:**

We analysed transcripts from video recordings of clinical interactions between patients with Stage III or IV cancer (n=26) and their oncologists (n=9). In‐depth discourse analysis was conducted to describe and interpret oncologists’ communication behaviours and common linguistic features in the interactions.

**Setting and participants:**

Data were from a larger study of patient‐provider communication between African Americans and oncologists at two cancer hospitals in Detroit.

**Results:**

Prognosis was discussed in 73.1% (n=19) of the interactions; treatment goals were discussed in 92.3% (n=24). However, analysis revealed that oncologists’ description of prognosis was vague (e.g. “*prognosis is a bit worse in your case*”) and rarely included a survival estimate. Oncologists often used ambiguous terminology, including euphemisms and jargon, and emphasized uncertainty (e.g. “*lesions are suspicious for the disease”*). Conversation about prognosis was frequently brief, moving quickly to the urgency and details of treatment.

**Discussion:**

This study demonstrates how oncologists’ language may obscure discussion of prognosis and treatment goals. The identified behaviours may lead to missed opportunities in eliciting and discussing patients’ knowledge about and preferences for their care. Patient‐, provider‐ and system‐oriented interventions are needed to improve clinical communication, especially among minority patients with advanced cancer.

## 1 INTRODUCTION

A recent report by the National Academy of Medicine concluded that cancer care delivery in the United States is in crisis.^1^ The care that patients receive is often not as patient‐centred, accessible or coordinated as it could be. Many of these problems apply especially to patients with advanced cancer. Numerous recent studies have shown that aggressive treatments continue to be widely administered to patients who are not adequately informed of the status of their cancer or the goals of their treatment. Moreover, despite the well‐documented benefits of palliative care—from improved symptom management and psychosocial outcomes to overall quality of life, and even longer survival time—there is a pervasive underutilization of palliative care for advanced cancer patients.^2^, ^3^, ^4^, ^5^ This underutilization is greater among minority individuals, relative to White patients.^6^, ^7^, ^8^, ^9^

Among the myriad reasons for suboptimal care provided to patients with advanced cancer, poor‐quality patient‐provider communication is recognized as a major factor.^10^, ^11^, ^12^, ^13^ Indeed, despite the National Academy of Medicine's recommendation that cancer patients be informed of their prognosis to enable them to make informed treatment decisions, few patients actually receive this information.^1^, ^13^ When such conversations do occur in clinical encounters, they have been observed to be problematic in that they do not achieve the goal of patient understanding of prognosis. For example, in a UK study with patients participating in Phase I Clinical Trials, Jenkins et al.^13^ observed that prognosis was only discussed in 21% of conversations (n=52) and physicians rarely checked patient understanding of prognosis. Compounding the difficulties in communication about prognosis, patient‐provider communication tends to be worse (e.g. less information exchanged) for ethnic/racial minority patients, including African Americans in the United States.^6^, ^9^, ^14^, ^15^, ^16^, ^17^ Similar patterns hold for communication at the end of life.^6^, ^18^, ^19^ Poor communication quality may be a factor in the well‐documented health disparities in cancer treatment and outcomes.^16^, ^19^, ^20^ Hence, in‐depth investigation into the way prognosis and goals of care are communicated during clinical interactions with African American patients with advanced cancer is especially warranted.

Recognizing the central role of patient‐provider communication in cancer care, social scientists over the last decade have endeavoured to better understand oncology prognosis discussions—including their barriers and facilitators—using a variety of approaches. For example, Leydon (2008) qualitatively analysed oncology consultations about cancer treatment options, focusing on the role of conveying optimism in light of uncertainty or “bad news”.^21^ Many similar communication studies have been done on prognosis conversations. For example, in an observational study of clinical interactions, Robinson et al. analysed transcripts of 141 interactions between oncologists and patients with advanced cancer.^22^ They found that while treatment was discussed in the majority of interactions (94%), prognosis (defined as “a statement about expectations of the disease that refer to the likely course of the patient's cancer or what the outcome might be”) was discussed in only 50% of the interactions. They also examined features of the oncologists’ communication behaviours that correlated with patient‐provider concordance in understanding of the chance for a cure: when oncologists made at least one pessimistic statement, patients were more likely to agree with their oncologist's estimated chance of cure. On the other hand, statements of optimism and uncertainty were not associated with an increased likelihood of concordance. Finally, in the context of medical interviews in which a patient's death and dying are discussed, a Conversation Analysis project described the contribution of the patient's responses to the oncologists’ delivery of the news about patients’ poor prognosis.^23^

In addition to observational studies, researchers have analysed patient interviews and other self‐reported data in order to better understand how patients are informed about prognosis during clinical interactions. For example, Step and Ray conducted in‐depth interviews with 30 female cancer patients about their experience with prognosis conversations during initial diagnosis and cancer recurrence.^24^ Patients reported that initial diagnosis conversations included physicians communicating optimism about prognosis, but during recurrence, the conversations shifted to a focus on the logistics of disease management. During recurrence conversations, much more uncertainty was communicated, leading to tension‐filled “prognosis dance” where patients and physicians both seek and avoid information^24^ (p. 54‐55). Finally, palliative care practitioners and educators have attempted to describe key problems in prognosis discussions and offer practical guidelines to improve discussions in clinical practice.^10^, ^25^, ^26^

This paper builds upon prior observational research by providing an in‐depth examination of oncologists’ language in real‐life medical encounters with African Americans with advanced cancer. Discourse analysis, a qualitative approach to analysing interactions, is useful in this context because it is grounded in theories derived from linguistics, communication science, psychology and sociology and may help researchers identify common behaviours employed by oncologists during sensitive encounters and to interpret and explain these behaviours.^27^, ^28^, ^29^, ^30^ This analysis serves to further explain factors that may contribute to patients’ poor understanding of the status of their disease. The findings from this research can inform the assessment of oncologist communication, as well as the development of interventions. The present analysis aims to describe oncologists’ communication behaviours and language **during initial discussions about prognosis and treatment goals** with a small sample of African Americans with advanced breast, lung, or colorectal cancer.

## 2 METHODS

**Study Data** were taken from a larger National Cancer Institute‐funded study conducted from 2012 to 2014, designed to improve patient‐oncologist communication during racially discordant interactions in the outpatient clinics of two cancer hospitals in Detroit, Michigan, USA.^31^, ^32^ Oncologists were eligible to participate if they treated patients with breast, colon or lung cancer. Patients (n=137) participated in the larger study if they self‐identified as Black, African American or Afro‐Caribbean, were between the ages of 30 and 85, were able to read and write English well enough to provide consent and answer the questionnaires, had a confirmed diagnosis of breast, colorectal or lung cancer and were scheduled for an appointment to see a medical oncologist to discuss medical treatment (e.g. chemotherapy). Thirty‐five of the 40 eligible oncologists (87.5%) agreed to participate, and of these, 18 (51.4%) had patients who participated and were included in the study sample.

The Institutional Review Boards of both hospitals and Wayne State University approved all procedures. Data from the present study consisted of transcripts of video recordings from encounters with all patients with Stage III (n=20) or IV (n=6) cancer, their family caregivers (when applicable), and their oncologists (n=9), as well as participants’ basic demographics and patient's medical information. These 26 encounters comprised the study sample. Mean length of encounters was 36.59 minutes (SD=15.74). Table 1 describes the participant characteristics.

**Table 1:** Patient and oncologist characteristics

Patients^a^	n=26
Age	M=58.58 (SD=13.63)
Female	20 (77%)
Education	
<High school	8 (31%)
Graduated high school	4 (15%)
Some college	7 (27%)
Graduated college	5 (19%)
Post‐graduate degree	2 (8%)
Annual household income	
0‐$19 999	9 (35%)
$20 000‐$39 999	8 (31%)
$40 000‐$59 999	3 (12%)
$60 000‐$79 999	1 (4%)
>$80 000	2 (8%)
Primary tumour site	
Breast	15 (58%)
Colorectal	5 (19%)
Lung	6 (23%)
Stage	
III	20 (77%)
IV	6 (23%)

Oncologists	n=10
Age	M=47.00 (SD=9.63)
Male	7 (70%)
Race/Ethnicity	
Caucasian or White	5 (50%)
Asian or Pacific Islander	3 (30%)
Arab American/Mideastern	2 (20%)
Attending (v. Fellow)	9 (90%)

a Some data are missing because of omissions in patients’ responses.

The following steps were followed to obtain study findings. First, our multidisciplinary team of investigators, including behavioural scientists and oncologists, reviewed all 26 transcripts to reach consensus on definitions of “prognosis” and “treatment goals” discussions. **Prognosis Discussion** was operationally defined as *any mention of the probable course or outcome of a disease, especially the chances of recovery*. **Goals of Treatment Discussion** was operationally defined as *any mention of why a treatment is being considered (e.g. to cure, treat symptoms or shrink the tumour)*. Two authors (LH, SE) then independently identified and extracted transcript excerpts in which discussions of treatment goals and prognosis occurred; the selected excerpts were then verified by two other authors (WSC and CT). Disagreement among team members was resolved through discussion.

Focusing on the extracted excerpts, we performed a qualitative discourse analysis, with the goal of identifying oncologists’ communication behaviours, as well as specific linguistic features related to prognosis and goals of treatment.^27^ The first author, a trained sociolinguist, identified prominent features that emerged and offered interpretations informed by theories of social interactions.^27^, ^29^ She and the study team then discussed preliminary findings and refined interpretations through an iterative process. In presenting the following qualitative findings, we use examples to highlight each particular linguistic feature, then draw on relevant literature (theories and descriptive studies) in order to discuss how the feature affects the consultation and discussion of prognosis. The examples/quotes below are prefaced by a patient identifier (letter A‐Z) and their diagnosis, namely cancer stage and site.

### 2.1 Findings

#### 2.1.1 Frequencies of prognosis and goals of treatment discussions

Prognosis discussions occurred in 19 (73.1%) of the interactions. Examples 1 and 2 illustrate some of the ways oncologists described prognosis:

**Example 1** (Pt G; Stage III CRC) *So if I put you in the Xerox machine and I add a hundred of you, okay, fifty of you would be cured with the surgery alone*.

**Example 2** (Pt D; Stage IV Breast) *HER2 is a receptor on breast cancer that actually means, if you're HER2 positive, it means that the prognosis is a bit worse than being negative, but the good news is that we do have a treatment for it*.

Example 1 represents a statement of prognosis, suggesting that half of patients with the same diagnosis can expect to be cured with surgery alone. This statement additionally offers the patient information about treatment decision making regarding surgery and other modalities. Example 2 contains a general and vague discussion, whereby the oncologist immediately downplays the “worse” prognosis with “good news,” referring to the treatment he is proposing. Such brief mention of prognosis followed by other information is typical of language used by oncologists in the study sample.

In contrast to prognosis, treatment goal discussions occurred in most (n=24, 92.3%) of the interactions:

**Example 3** (Pt C; Stage IV Breast) *So what the chemotherapy does is that it tries to attack those cells and prevent the cancer from coming back*.

In this example, the oncologist explains how chemotherapy works and what its goals are, as part of the discussion of a treatment plan. Such discussions occurred frequently in the sample. The next section will explore key strategies and features commonly observed, with the goal of shedding light on how these sensitive discussions are managed or minimized.

#### 2.1.2 Oncologists’ discussion of prognosis and treatment goals: Macro‐level communication strategies and micro‐level linguistic features

Our analysis was informed by a discourse analytic framework, which allowed us to distinguish between macro‐level communication strategies and micro‐level linguistic features. Broad communication behaviours (e.g. interruption or topic avoidance) are considered to be macro‐level communication behaviours that are realized by specific micro‐level linguistic features (e.g. use of a certain pronoun or word). For a comprehensive discussion of function‐form connections, see Johnstone's text on Discourse Analysis.^27^

The analysis identified three macro‐level communication behaviours in oncologists’ discussion of prognosis and goals of treatment: (1) minimizing prognosis discussion, (2) stressing uncertainty and (3) emphasizing hope. Additionally, we identified three micro‐level linguistic features that comprise these communication behaviours: (1) euphemism/ambiguity, (2) modal expressions and (3) jargon. Each of these observed strategies is summarized below, beginning with a description and definition, followed by transcript illustrations and interpretations based on relevant theories.

### 2.2 Macro‐level communication strategies

*(1) Minimizing prognosis discussion* by moving quickly to treatment goals/plans or merging prognosis with goals of treatment talk.

An in‐depth transcript review showed that prognosis discussions were often brief. Oncologists often quickly referenced diagnosis and moved directly to extensive discussions of treatment plans and associated logistics. As demonstrated in Example 4, the oncologist discloses the tumour's growth and location and moves immediately, without a pause, to a detailed description of a treatment plan (not shown):

**Example 4** (Pt V; Stage IV CRC) *Right below the pubic bone, the hip bone over here, your tumor has grown quite large, about twice or three times larger, that means you need treatment, okay?*

This observed sequence of topics differs from the typical “routine” structure of medical encounters, as described by Ten Have's conversation analysis of medical consultations: opening‐>complaint‐>examination‐>diagnosis‐>treatment/advice‐>closing.^30^ Ten Have posits that a diversion from this typical sequence is marked as it runs counter to the expectations of the participants. Upon mentioning that the cancer has grown, the oncologist leaves no time to discuss the cancer diagnosis and its implications for a poor prognosis. Instead, he launches into the next topic—the urgency of treatment. This type of switch from referencing a poor prognosis directly to treatment planning is very common in these data, whereby oncologists either move quickly from “diagnosis” talk into “treatment/advice” talk or merge the two.

*(2) Emphasizing uncertainty and lack of information*

In discussing prognosis and goals of treatment, oncologists were observed to emphasize the fact that they did not have sufficient information about the patient's disease. Example 5 illustrates a typical discussion focusing on future tests and information gathering:

**Example 5** (Pt E; Stage IV Lung) *Now I can go with a different chemo but I feel it might not be a bad idea to repeat your scans in 3 weeks and see what the scans look (like). If it looks like things are still stable and aren't growing, we may just give you some time off…*

The oncologist devoted most of the encounter to discussing future tests and the diverse implications of potential results. Han et al.'s taxonomy of types of uncertainty in health care provides a helpful framework in understanding the role of discussing future tests.^33^ Considering the range of examples of uncertainty outlined in Han et al., both scientific (data‐centred) issues (diagnosis and treatment recommendations) and practical (systems‐centred) issues (process towards getting more diagnostic workups) are referenced here.^33^ The discussion of uncertainty remains very scientifically oriented. In contrast to the inherent uncertainty of future test results, what is actually known—namely, an advanced, Stage IV lung cancer diagnosis—is barely discussed. The frequent emphasis on uncertainty and the need for more information may potentially hinder patients’ understanding of prognosis and goals of treatment. It is possible that this emphasis on uncertainty might detract from conversation about what is known.

*(3) Emphasizing optimism and hope*

Oncologists were observed to emphasize hope when a potentially poor prognosis was indirectly referenced:

**Example 6** (Pt L; Stage III Lung) *And I will tell you this is frankly a bit on the outer edge of our ability to get rid of. I want to be very candid with you. But I do believe that we can do this*.

This oncologist referenced a poor prognosis, namely, the limited chance of a cure. However, right after admitting “being candid” about the prognosis, he established his belief in “doing this” —what “this” entailed was unclear, but a sense of optimism in the treatment was conveyed. Expressions of optimism in patient‐provider encounters have been studied. For example, Robinson et al. examined the association between physicians’ expressed optimism and patients’ and providers’ alignment in prognostic understanding.^22^ The researchers found that physicians’ expression of optimism is linked to misaligned understanding of prognosis. It is possible that a focus or orientation towards possible effective treatments and a sense of optimism, though well intentioned, contribute to confusion or misunderstanding about the diagnosis and prognosis.

### 2.3 Micro‐level Linguistic features

How are the above‐mentioned communication strategies achieved linguistically? A discourse analysis identified three micro‐level linguistic features; each of which will be described and defined below, along with illustrative examples and a theory‐based interpretation.

*(1) Use of euphemism and linguistic hedging*

Oncologists were observed to broach the topic of prognosis indirectly. One common way of signalling indirectness is through the use of euphemistic expressions. Euphemisms are a kind of linguistic hedging which functions to signal the speaker's sense of uncertainty towards a proposition and downplay their epistemic stance towards a statement.^28^ For example, words such as “lesion,” “spot” and “stuff” were used frequently to reference cancer, and a generic and technical term, “activities,” was used to reference cancer growth.

**Example 7** (Pt I; Stage III Lung) *So I want to make sure it's* *abnormal tissue* *. It's a* *mass* *or* *lesion* *or* *tumor* *or…*

The oncologist brings up a number of alternative terms—from “abnormal tissues” to “mass,” “lesion” and “tumour” —none of which offers a definitive diagnosis or prognosis, adding to ambiguity and potential for confusion. In addition to these generic referring terms for cancer, there are other euphemistic expressions in the language of the oncologists.

**Example 8** (Pt B; Stage IV CRC)
Dr: *So the surgeons wanted you to come see me as a medical oncology doctor so that we could start* *treating* *the cancer with chemotherapy in the hopes of* *shrinking* *everything* *so that they could then reevaluate you*
Pt: *Right…*
Dr: *And see where else, you know, what else* *they could do to try to* *,* *you know* *,* *improve your chances*.

The oncologist in Example 8 touches upon the goals of chemotherapy treatment as “shrinking everything” for further evaluation. He does not reference the current status of the cancer directly; however, from the discussion, one might infer that it is inoperable due to the size. The generic noun “everything” indirectly refers to the cancer. Note also that the verb “treat” here does not entail a curative measure, but merely helps to reduce the size of the tumour to make surgery possible as a subsequent treatment option. Finally, the phrase “improve your chances” again indirectly suggests a grim prognosis, and this indirectness is further shown with linguistic hedges such as “could” and “try to.” The discourse marker “you know,” uttered twice by the oncologist solicits the patient's affirmation of the receipt of previously mentioned information and possibly signals the speaker's sense of uncertainty towards the proposition.^29^ Linguistic hedging serves to convey ambiguity; for example:

**Example 9** (Pt L; Stage III Lung) *The hope is that the total package gets rid of this…but whether we will or not only time will tell*.

In discussing the goal of the treatment and its potential effectiveness, the oncologist's language sets up “hope” but then leaves the prospect of getting rid of cancer uncertain with the phrase “only time will tell.” This language could potentially leave the patient confused about the goal of treatment and likelihood of it being effective.

Taken together, the frequent use of euphemisms and hedging to discuss prognosis suggests that oncologists may find that directly addressing this sensitive topic to be *face‐threatening,* and hence mitigate this threat through language. This explanation would align with the framework of face^34^ and politeness theory.^35^ These theories posit that in social interactions, including and particularly face‐to‐face exchanges, in addition to conveying information, speakers have to constantly mitigate potential “face threats” (i.e. threats to a desired self‐image) carried by certain face‐threatening acts towards another individual. Talking to a new patient directly about a life‐threatening diagnosis and their mortality is inherently face‐threatening, and naturally oncologists find strategies to mitigate the uncomfortable discourse, often through indirect speech.

*(2) Use of Modal expressions*

Indirectness in the oncologists’ language can also be observed by their use of modal expressions such as conditionals, especially in the discussion of prognosis.

**Example 10** (Pt D; IV breast)
**Family Member:** *Now if we're at a Stage four, then what's the life expectancy?*
Dr: *So stage four patients, you know, the average life expectancy at the time of diagnosis is two years, but fifty percent of patients, they live more than two years and fifty percent less*. *If there is only cancer in bones, for example, I have patients who have lived for ten years or so because, you know, cancer is only in bones. But if it's in lungs, you know, it's kind of worse, but on average, it's two years*.

In response to the family member's question about prognosis, the oncologist suggests that fifty percentage of patients with Stage IV lung cancer will live more than two years, followed by the use of a series of conditional expressions (“if…”) to discuss different possible prognostic outcomes depending on whether the cancer is only in the bones or also in the lungs. The discussions are inherently modal: modality in linguistics refers to “expressions that relate to potentially unreal situations”.^36^ It is impossible for the patient and doctor here to know exactly whether the cancer has metastasized to the bones. Bringing out alternative scenarios through modal expressions contributes further to uncertainty and confusion about the prognosis, which is typically poor for a Stage IV diagnosis.

Later on in the same encounter, the doctor continues the discussion of the prognosis in Example 11 through conditional expressions (“If…”) and hedges (“we think, we believe”).

**Example 11** (Pt D; Stage IV Breast) *… we think, we believe that the cancer is only in the breast and maybe in the lymph nodes, but if the cancer cells have escaped from the lymph node and if they have spread to the other parts of your body, it means that we can't cure you. It means that the cancer is basically, you will die of breast cancer, but we have a lot of treatments still…*.

Here, the oncologist touches upon the potential of dying from cancer, but minimizes the possibility by providing many hypothetical scenarios. This is typical, wherein oncologists used modal expressions and listed alternative prognostic scenarios (some of which were unreal) as a way to minimize the potential for a poor prognosis.

*(3) Use of Complex language and medical jargon*

Use of complex language and medical jargon were observed frequently. Such complex language was evident in both the vocabulary choices and in the syntactic structures of the oncologists. For example, examples 12‐13 illustrate technical expressions:

**Example 12** (Pt A; Stage IV Lung) *But, if everything is shutting down and everything is responding, then I'll say well it is because of* *systemic disease* *. That's great that you responded. We will give more chemotherapy, but we will not give radiation…As well as radiation would be good sign, that means disease was* *localized* *. If we cannot give radiation that means that it most likely was* *systemic* *and we just proceed with* *systemic chemotherapy*.

**Example 13** (Pt L; Stage III lung) *It has* *features of both types* *of cancers. It's still one cancer, but it has* *features of both* *cancers and usually the type of non‐small lung cancer in those mixed tumors is the* *squamous‐cell sub‐type*.

The explanation in Example 12 is complex and includes terms (underlined) that are not likely understood by laypersons. Moreover, the descriptions of the cancer types and the technical use of the word “feature” in Example 13 represent a techno‐scientific perspective. Complex language can extend beyond vocabulary/lexical level to the syntactic level, whereby the use of complex sentence structures presents additional challenges to patient understanding.

**Example 14 (**Pt A; Stage IV Lung**)** *We still don't know what that two nodules…means…. I think* *potentially* *disease is only in the lung, although these 2 lesions are kind of* *suspicious for the disease*.

In addition to descriptions such as “these lesions are kind of suspicious for the disease,” the complex language use potentially causes more ambiguity and confusion during the discussion of high‐stake topics including prognosis and goals of treatment.

## 3 DISCUSSION

This study focuses on prominent linguistic features in oncologists’ language in discussing prognosis and treatment goals with patients with advanced cancer. This analysis revealed ways in which the oncologists’ use of language may have contributed to a lack of adequate or effective discussion of prognosis and goals of treatment, and thus potentially prevented informed decision making. This study also illustrates the value of direct observations of oncology encounters that allow us to highlight characteristics of participants’ linguistic strategies and features. We identified several missed opportunities for more in‐depth discussions about prognosis and goals of treatment. Given the inherent uncertainty and high‐stake nature of oncology encounters and the demonstrated importance of accessible clinician language,^18^, ^37^ the observed ambiguous language may potentially exacerbate the problem of poor understanding of prognosis and goals of treatment and may distract from, or preclude, a full exchange of information.

The analysis begs the question: why is effectively communicating prognosis and goals of treatment so difficult? On the patient's side, there are tremendous variations in information preferences when facing an advanced cancer diagnosis.^38^, ^39^, ^40^ Some patients and family members are reluctant to discuss prognosis and may block such discussion from taking place; others may experience cognitive and emotional overload and physical suffering, preventing them from attending to these difficult topics. On the provider's side, reasons may include concerns about protecting the patient's and provider's “face”^35^ and inadequate clinical communication skills training and coaching.^41^, ^42^, ^43^ Also, pending further study, the oncologists’ communication behaviours may have been influenced by clinician bias or mistrust and concerns coming from the patients.

Over the last decade, clinicians' communication skills have gained tremendous attention in medical education, as reflected in new medical training curricula and requirements.^44^, ^45^, ^46^ This linguistic analysis offers promising directions to pursue in attempt to improve oncologists’ clinical communication by way of paying attention to and possibly altering their language when talking with patients. One potential strategy that may improve oncologist‐patient communication calls for integrating linguistic insights into existing training curricula. For example, a holistic mindful communication training has demonstrated benefits for clinicians, whereby trainees reported improvements in well‐being and attitudes associated with patient‐centred care.^47^, ^48^ As a next step, it may be possible to integrate insights about language into such a curriculum, for example, illustrating the above communication strategies and linguistic features (e.g. the use of euphemisms or ambiguity expressions) to clinician trainees. Clinicians may benefit from being made more aware of the common linguistic features that may contribute to confusion or ambiguity. We can evaluate whether drawing attention to nuances of language by providing video‐recorded examples for physicians to observe and reflect upon, may change communication behaviours, improve prognostic discussion and facilitate informed decision making.

Methodologically speaking, our qualitative discourse analysis complements other observational and survey methods frequently used to study patient‐provider communication. A focus on language allows us to dive deeper into the sequential unfolding of interactions and provides partial explanations for why these exchanges often lack key aspects of the prognosis and goals of treatment. In essence, we observe a common trajectory of interaction focusing on future events that may be unlikely to provide a cure.

*The limitations of this study* include the fact that even though the interactional context is taken into account in our qualitative study, the central focus is on the oncologist and their communication behaviours. In addition, we only analysed transcripts; therefore, we were unable to account for non‐verbal behaviours in these encounters. The dynamic, interpersonal process of clinical communication about prognosis warrants further investigation.^49^ Future research should conduct dyadic and triadic analyses on these interpersonal communication processes, as well as focusing on patient behaviours. Another limitation is the fact that this is a small sample taken from one patient population from one geographic location, and thus, findings may not be generalizable. However, this is one of the first studies to conduct this type of in‐depth, descriptive analysis of clinical interactions with African American patients. This population bears a disproportionate burden of cancer disparities including incidence, mortality, communication and care.^20^, ^50^

## 4 CONCLUSION

This study examined oncology communication in action through an in‐depth qualitative linguistic analysis of real‐life, real‐time clinical encounters with patients diagnosed with advanced cancer. Through discourse analysis, we identified a number of oncologists’ communication behaviours and described how they fit into the overall consultation genre,^30^ in order to better understand how sensitive topics such as prognosis and goals of treatment are (or are not) discussed. This type of in‐depth analysis complements counting/coding studies, allowing for a description and interpretation of not only what is discussed, but what is *not* discussed. In addition, the study points to next steps in research, including in‐depth interviews with patients, caregivers and providers to complement observations and triangulation across multiple sources of data (e.g. care utilization data or secondary surveys). With rapid advances in cancer treatment and promising new drugs being developed, it is likely that prognostic information will only become more confusing and uncertain. This study has the potential to inform communication training programmes, helping providers to be more mindful of their language use in order to effectively convey high‐stake information such as prognosis and goals of treatment to their patients.
